# Psychometric properties of a new intake questionnaire for visually impaired young adults: The Participation and Activity Inventory for Young Adults (PAI-YA)

**DOI:** 10.1371/journal.pone.0201701

**Published:** 2018-08-07

**Authors:** Ellen Bernadette Maria Elsman, Gerardus Hermanus Maria Bartholomeus van Rens, Ruth Marie Antoinette van Nispen

**Affiliations:** 1 Department of Ophthalmology, Amsterdam UMC, Vrije Universiteit Amsterdam, the Amsterdam Public Health research institute, De Boelelaan, Amsterdam, the Netherlands; 2 Department of Ophthalmology, Elkerliek Hospital, Helmond, the Netherlands; University of Maiduguri College of Medical Sciences, NIGERIA

## Abstract

**Background:**

To be able to identify and monitor personal needs and goals of visually impaired young adults before and during rehabilitation trajectories, the Participation and Activity for Young Adults (PAI-YA) was developed involving young adults (18–25 years) and professionals as stakeholders. The psychometric properties of this new patient-reported outcome measure were investigated in order to develop an improved version.

**Methods:**

Young adults registered at two low vision rehabilitation centers in the Netherlands were invited to complete the 141-item PAI-YA (n = 186) in a test-retest design. To select the best items for the PAI-YA, response frequencies were assessed and a graded response model (GRM) was fitted. Item reduction was informed by response frequencies, insufficient item information, and participants’ comments. Fit indices, item and person (theta) parameters were computed, after which known-group validity, concurrent validity, test-retest reliability and feasibility were studied.

**Results:**

Response frequencies, violation of assumptions and item information informed the elimination of 81 items, resulting in a unidimensional PAI-YA showing satisfactory fit to the GRM. Known-group validity showed significant differences for visual impairment, financial situation, sex, educational situation and employment situation. Concurrent validity with (scales of) other questionnaires showed moderate to strong expected correlations. Test-retest reliability was satisfactory for all items (kappa 0.47–0.87), as was agreement (63.1–92.0%). Four items and one response option were added to increase feasibility.

**Conclusions:**

This study contributes to the development and assessment of psychometric properties of the PAI-YA, which resulted in an improved 64-item version. Evidence was provided for construct validity, known-group validity, concurrent validity and test-retest reliability. These results are an important step in the development of a feasible instrument to investigate and monitor rehabilitation needs of visually impaired young adults, to structure the intake procedure at low vision rehabilitation services and to evaluate the effectiveness of rehabilitation.

## Introduction

The prevalence of visual impairments among young adults aged 18–25 years in the Netherlands is low, affecting approximately 0.1–0.2% of the population [1]. However, having a visual impairment has a major impact on daily functioning and quality of life [2–5]. Furthermore, persons with low vision report higher prevalence of loneliness and experience more participation restrictions [6, 7]. Young adults face important life transitions, characterized by the longing for increased independence and autonomy [8]. Various developmental tasks and goals are associated with the transition to adulthood, e.g. completing school, gaining employment, living independently and selecting a partner [9–13]. Having a disability can make this transition highly challenging and might interfere with goals associated with the transition, causing psychological distress [14] and disruption in the pursuit of one’s developmental tasks [15]. For example, although young adults with a visual impairment are less often employed compared to their sighted counterparts [16], this cannot be explained by differences in dropout and graduation rates [16, 17]. Furthermore, they experience difficulties in establishing and maintaining social, intimate and romantic relationships, which may threaten psychosocial development [9–12, 18, 19]. In addition, these individuals are more likely than their sighted peers (or persons with other disabilities) to live with their family after finishing school [20–22]. Multidisciplinary rehabilitation centers (MRCs) for the visually impaired can play a role in identifying the difficulties young adults experience, and offer guidance and rehabilitation to help overcome these difficulties [23].

In the Netherlands, MRCs use the Participation and Activity Inventory (PAI, formerly known as the D-AI) to identify personal goals of adult clients [24]. This questionnaire was developed and implemented in the Netherlands and originates from the Activity Inventory created by Massof et al. [25]. The PAI consists of the nine domains of the Activity and Participation component of the International Classification of Functioning, Disability and Health (ICF) from the World Health Organization [26, 27], and was found to be feasible and to have sound psychometric properties [28, 29]. The PAI can be used to identify needs, set goals and create an individualized rehabilitation plan. Moreover, the PAI provides insight into the effectiveness of rehabilitation and can be considered a patient-reported outcome measure (PROM) [30, 31].

Currently, the PAI is used for all adults in Dutch MRCs. However, the life stage of young adults aged 18–25 years is characterized by the transition to becoming an adult, making the extensive content of the PAI less applicable for this particular population [32]. Therefore, the PAI is less often used during the intake of young adults, who often receive a semi-structured interview instead [5, 32], increasing the risk of bias and overlooking specific needs, especially in domains which are not straightforward (e.g. relationships, and recreation/leisure). Furthermore, incorrect identification of needs might influence referral to rehabilitation programs and the quality of care provided [33]. To overcome this, a preliminary version of the PAI for Young Adults (PAI-YA) has recently been developed using a concept-mapping procedure, and has been further improved in a pilot study to assess its feasibility [5, 34].

The concept-mapping procedure and pilot study contributed to the face and content validity of the PAI-YA. The aim of the present study is to assess the psychometric properties of the PAI-YA and to develop an improved version.

## Methods

### The PAI-YA

The preliminary version of the PAI-YA comprises 141 items, which are grouped into 17 domains which were informed by the concept-mapping procedure: reading and visual aids (RV-5 items), mobility (MO-16 items), computer skills (CS-8 items), living independent and finances (LF-8 items), household (HH-7 items), self-care (SC-7 items), leisure time (LT-11 items), holiday and going out (HG-11 items), social relationships (SR-11 items), intimate/romantic relationships (RR-3 items), peer contact (PC-6 items), communication (CO-11 items), information/regulations (IR-12 items), study (ST-8 items), applying (AP-4 items), work (WO-6 items), and acceptance/self-consciousness (AS-7 items).

Each item is scored on a 4-point Likert scale with response options: not difficult (1), slightly difficult (2), very difficult (3), and impossible (4). The response option ‘not applicable’ is treated as a missing value. After each domain a question is asked to clarify the rehabilitation needs (Do you have any questions for the rehabilitation center regarding the topic … or would you like to receive rehabilitation for this?).

### Study design and participants

Young adults aged 18–25 years who were enrolled for care at two Dutch MRCs (Royal Dutch Visio, and Bartiméus) at the time of this study or in the past, were invited to participate in the present study. Participants had to have adequate knowledge and understanding of the Dutch language and sufficient cognitive ability to participate in the study. Young adults with registered extensive cognitive impairment were excluded from the selection of young adults to be invited to participate by the low vision rehabilitation centers. Young adults with low vision from any cause were eligible and there was no restriction regarding visual performance. All potential young adults were sent a letter explaining the aim, procedure and duration of the study and asking whether they would agree to participate. Young adults who did not respond were telephoned to further inform them about the study and ask for their reasons not to participate.

Young adults who accepted to participate in the study were also telephoned in order to explain the aim, procedure and duration of the study again, and a researcher assessed through conversation whether they had sufficient cognitive abilities to administer the questionnaire. Participants were asked to fill in a socio-demographic questionnaire, the PAI-YA, and a self-constructed evaluation form. They were also asked to fill in Dutch versions of comparator instruments to assess (vision-related) quality of life and participation. Young adults had the option to fill in the questionnaires through a web-based survey questionnaire, a paper-and-pencil version, a telephone interview, or a face-to-face interview (home visit), whatever option was most convenient to them. Two weeks after initial completion, young adults were asked to fill in the PAI-YA and evaluation form again, using the same mode of administration. This time interval was chosen because participants probably have remained stable, but are unlikely to remember their previous answers because of the length of the PAI-YA [35]. Participants with an excessive number of missing responses in the PAI-YA (over 50) were excluded from analysis.

The study protocol was approved by the Medical Ethical Committee of the VU University Medical Centre, Amsterdam, the Netherlands. This study was performed in accordance with the ethical standards as laid down in the Declaration of Helsinki. Written informed consent was obtained from all included participants.

### Other instruments administered

To assess concurrent validity of the PAI-YA, participants were asked to fill in four other instruments during the first administration. Two generic health-related quality of life instruments were administered, the Euroqol-5 Dimensions (EQ-5D) [36, 37] and the Short Form Health Survey (SF-36) [38]. The EQ-5D consists of five dimensions of functional impairment, whereas the items of the SF-36 can be assigned to eight scales. One instrument to assess participation was administered: the Impact on Participation and Autonomy questionnaire (IPA) [39], which consists of 32 items that can be assigned to four scales. Finally, one vision-related quality of life instrument was administered, the Low Vision Quality of Life questionnaire (LVQOL) [40], of which the 18-item unidimensional version validated by Van der Aa et al. was used [41]. For each instrument, a (scale)score was calculated following standard scoring rules [38, 40, 42–44]. Except for the EQ-5D, which is a formative scale, internal consistency reliability was assessed in the study population using Cronbach’s alpha for (subscales of) all instruments.

### Item selection

All analyses were performed using SPSS version 22 [45] and R using the ltm package [46]. Demographic variables were analyzed using descriptive statistics. Acceptability of the PAI-YA was determined by looking at response rates for each individual item and the evaluation forms. Item reduction occurred through a structured approach, in which emphasis was placed on creating an instrument with reliable measurement properties while maintaining content validity. In order to create a reliable and valid questionnaire, items for potential deletion were chosen using an iterative process consisting of item analysis and application of IRT.

#### Item analysis

Items with missing scores >40% were eliminated immediately from further analysis, while items with missing scores 20–40% were considered for elimination. Variability of the PAI-YA scores was assessed for each item using floor effects (percentage of respondents scoring at minimum level, i.e. (1) not difficult) and ceiling effects (percentage of respondents scoring at maximum level, i.e. (4) impossible). Floor and ceiling effects of items were considered to be present if >70% of the participants chose the lowest or highest possible response option. Item-pairs were flagged when inter-item correlation was >0.7, indicating that these items were similar and one of them is potentially redundant.

#### Application of IRT

IRT is a collection of modeling techniques from modern measurement theory which provides a powerful framework to build instruments which are more efficient, reliable and valid [47]. IRT represent a number of statistical models that describe the association between a person’s ability (latent trait) and the probability of a person to choose a certain response option of an item in a multi-item scale [48]. The graded response model (GRM) is one of the most commonly used polytomous models to evaluate questionnaires with ordinal response categories, and it estimates a discrimination/slope (α) parameter and extremity (β) parameters [49, 50]. Application of IRT requires three assumptions:

- One of the critical assumptions of IRT is unidimensionality, i.e. a single latent trait in the PAI-YA such as “participation” explains the covariance of items [47]. To investigate unidimensionality, principal component analysis (PCA) was conducted. The number of factors was assessed using a scree plot and acceleration factor, which uses a numerical solution for determining the coordinate where the slope of the curve changes most abruptly [51].
- Items should display local independence, i.e. item responses are independent given their relationship to the latent trait [47]. Local independence was assessed by inspection of possible excess covariation (>0.25) among items in the residual matrix resulting from PCA. Item pairs which held excess covariation were considered candidates for deletion; the least performing item was selected.
- Monotonicity means that the chance a respondent endorses a successive threshold on the response scale is larger for those with a higher latent trait score [52]. The monotonicity assumption was evaluated by assessing manifest monotonicity using Mokken scale analysis. Items with non-monotonic increasing graphs were considered candidates for deletion. Moreover, Loevinger H coefficients [53] were calculated to assess scalability of items (see also [54, 55]). The coefficient is calculated as a function of Guttman errors between item pairs. A Loevinger H coefficient of <0.30 is considered unsatisfactory, and these items were candidate for deletion.

After checking IRT assumptions, IRT analysis was conducted to assess model fit and identify items contributing little information. Basic model fit of the GRM was assessed comparing a full model [47] with a constrained model [47, 56], which is nested within the full model and has equal slope parameters across items (analogous to the Rasch model). A Likelihood Ratio test was conducted to assess whether the full model outperformed the constrained model. Subsequently, functioning of items was initially assessed by examining item information in the latent trait measured. Item information refers to the information content of an item in relation to the total test information. Therefore, information represents reliability or measurement precision [47]. Items with low information (initially contributing <0.75% of total information) across the disability continuum were considered for elimination. The Item Information Curves (IICs) and Category Response Curves (CRCs) were evaluated to support decision making. The IIC shows the range of the underlying trait over which an item is most useful to distinguish between participants. The CRC shows the relationship between the latent trait and the probability of responding to a categorical item (i.e. it displays the most likely categorical response across the latent trait). When items had similar curves, the one with least information and/or holding information over the smallest range of the latent trait was considered for elimination. Items flagged by the item analysis, evaluation of assumptions and IRT analysis were potential candidates for deletion. However, previous qualitative studies [5, 34] and comments made by young adults were also taken into consideration, as well as the researchers’ opinions.

### Psychometric properties of the PAI-YA

After the item selection process was completed, the reliability and validity of the PAI-YA was assessed. First, overall fit of the IRT model was assessed using the mirt package [57] yielding the M2 statistic and several fit indices: root mean square error of approximation (RMSEA) [58], standardized root mean square residual (SRMR), comparative fit index (CFI) [59] and Tucker-Lewis index (TLI). The CFI and TLI should be around 0.95 or higher, whereas the SRMR should be around 0.08 or lower and the RMSEA around 0.06 or lower [60]. The test information curve of the PAI-YA was presented, which refers to the underlying trait range over which an instrument is most useful to distinguish between participants. Subsequently, a person-item map was plotted for the items of the PAI-YA using the WrightMap package [61] to evaluate whether item difficulty matches respondents’ ability. The person-item map shows the distribution of person parameters (thetas) of participants on the left side of the map, and the distribution of item thresholds on the right side. Next, known-group validity was assessed by analyzing differences in thetas between relevant groups to reassure the PAI-YA can differentiate between groups: visual impairment, sex, age, nationality, mode of administration, financial situation, cognitive impairment, comorbidity, education in years, educational situation, and employment situation. Thetas of relevant groups were compared using independent samples t-test, ANOVA with posthoc Tukey-tests for multiple inter-group comparisons and Spearman’s correlation. Significant differences were expected for severity of the visual impairment, financial situation, comorbidity, educational status and employment status. To correct for other variables, multiple linear regression including all variables was performed. Concurrent validity was assessed by investigating the associations between the PAI-YA and (scales of) the SF-36, EQ-5D, LVQOL and IPA using Spearman’s correlation. Negative correlations were expected between the PAI-YA and (scales of) the SF-36, EQ-5D and LVQOL. Positive correlations were expected between the PAI-YA and scales of the IPA. Moderate (i.e. 0.3–0.5) to strong (i.e. >0.5) correlations [62] were expected between the PAI-YA and scales of the SF-36 and IPA, and strong correlations between the PAI-YA and the EQ-5D and LVQOL. Last, test-retest reliability of individual items was investigated using weighted kappa and percentage agreement. Kappa values >0.40 are considered moderate, >0.60 good and >0.80 very good [63]. Agreement is considered moderate for percentages of 60–74%, percentages of 75–89% are considered good and percentages ≥90% are considered excellent [64].

## Results

### Participant characteristics

Of the 1085 young adults invited for participation in the study, 218 (20.1%) agreed to participate and gave their written informed consent. Main reasons for non-participation were no time (31.7%), not interested (30.6%), and not able to participate because of cognitive impairment or not being visually impaired (15.5%), as indicated by data from non-responders with whom contact by telephone could be established. Of the respondents who agreed to participate, 193 completed the first PAI-YA questionnaire (88.5%). Seven participants were excluded from the analysis due to an excessive number of missing responses. Socio-demographic characteristics of the remaining participants are presented in Table 1. The retest was completed by 151 participants. Mean time between completion of test and retest was 30.58 ± 29.20 (range 11–171, median 18) days.

**Table 1:** Socio-demographic characteristics of participants (N = 186).

Age in years at completion date of the first questionnaire, mean ± SD (range)	21.56 ± 2.42 (16–30)
Male gender, N (%)	85 (45.7)
Self-reported vision loss ‡	
Blind, N (%)	30 (16.1)
Low vision, N (%)	89 (47.8)
Mild vision loss, N (%)	55 (29.6)
Unknown, N (%)	12 (6.5)
Education in years, mean ± SD (range)	11.34 ± 2.58 (0–16)
Method of completion	
Online, N (%)	162 (87.1)
Telephone interview, N (%)	22 (11.8)
Paper-and-pencil version, N (%)	2 (1.1)
Face-to-face, N (%)	0 (0.0)
Nationality	
Dutch, N (%)	170 (91.4)
Other, N (%)	16 (8.6)
Currently studying, N (%)	120 (64.5)
Currently having a paid (part-time) job, N (%)	65 (34.9)
Currently doing voluntary work, N (%)	45 (24.2)
Financial situation	
Usually enough money, N (%)	93 (50.0)
Just enough money, N (%)	55 (28.5)
Not enough money, N (%)	13 (7.0)
No answer, N (%)	27 (14.5)
Cognitive impairment	
No, N (%)	170 (91.4)
Yes, N (%)	8 (4.3)
Don’t know, N (%)	8 (4.3)
Comorbidity, N (%)	74 (39.8)

‡Blind: corrected decimal visual acuity ≤0.05 of the best eye; low vision: corrected decimal visual acuity ≤0.3 and >0.05 of the best eye; mild vision loss: corrected decimal visual acuity >0.3.

### Item selection

Table 2 shows the distribution of participants over the response categories for the PAI-YA items. Thirteen items had missing scores >40% and were eliminated from further analysis; 24 items had missing scores 20–40% and were considered for elimination. Infrequent endorsement of the response option “impossible” motivated collapsing the response options “very difficult” and “impossible”. Assessment of floor and ceiling effects indicated no ceiling effects, whereas floor effects were found in 37 items and these were reconsidered for inclusion. High inter-item correlations (>0.7) were found between eight item pairs, and one item of each pair was flagged for elimination.

**Table 2:** Distribution of responses over the response options, and phase and reason for item removal.

Item	Item content^a^	Missing response (%)	Distribution of responding population (%) over the response options				Phase and reason for item removal
			1	2	3	4	
RV1	Reading text	2.2	42.3	41.2	13.7	2.7	
RV2	Reading handwriting	3.2	22.2	41.7	26.1	10.0	
RV3	Reading braille	74.7					0: Missing responses
RV4	Finding visual aids	18.8	57.0	33.8	7.9	1.3	1: Information; local dependence
RV5	Using visual aids	18.8	64.9	28.5	6.6	0.0	
MO1	Walking	0.0	72.6	20.4	6.5	0.5	
MO2	Cycling	7.5	48.3	26.2	11.6	14.0	
MO3	Driving a scooter	51.6					0: Missing responses
MO4	Driving a car	46.2					0: Missing responses
MO5	Participating in traffic at daytime	1.6	60.7	32.2	4.9	2.2	
MO6	Participating in traffic at night	0.5	25.9	46.5	18.9	8.6	
MO7	Estimating the speed of other traffic	2.7	27.1	47.5	19.3	6.1	
MO8	Reading traffic signs	5.9	16.0	35.4	30.3	18.3	2: Information; local dependence
MO9	Handling steps and stairs	1.1	46.7	40.2	13.0	0.0	4: Local dependence
MO10	Traveling with public transport	0.5	53.0	34.1	10.3	2.7	
MO11	Finding support for independent travelling	30.1	69.2	22.3	8.5	0.0	1: Missing responses; comments YA
MO12	Finding the way to and at an unknown location	1.6	29.5	36.1	27.9	6.6	
MO13	Exploring the own neighborhood	1.1	75.5	20.1	3.3	1.1	3: Similar curves MO16 & CS6
MO14	Exploring a new neighborhood	1.6	38.8	36.1	20.2	4.9	4: Local dependence; inter-item correlation MO12 and HG3
MO15	Finding the way to school or work	8.1	81.9	15.8	1.2	1.2	
MO16	Finding the way at school and work	6.5	73.6	21.8	4.0	0.6	
CS1	Reading text from a screen	1.1	53.3	35.3	9.2	2.2	1: Information; comments YA
CS2	Banking online	3.2	77.8	13.9	6.1	2.2	1: Information; floor effect
CS3	Using digital visual aids	28.5	73.7	21.1	5.3	0.0	1: Missing responses; information; floor effect; comments YA
CS4	Finding suitable computer software	16.1	63.5	22.4	12.2	1.9	
CS5	Using the computer	2.2	82.4	15.4	1.6	0.5	
CS6	Finding suitable apps for tablet or smartphone	9.1	75.7	15.4	8.3	0.6	4: Local dependence
CS7	Using the tablet or smartphone	0.5	80.6	16.1	2.7	0.5	1: Information; floor effect; local dependence
CS8	Using social media	3.2	87.8	8.3	3.3	0.6	
LF1	Paying	1.6	74.9	22.4	1.6	1.1	
LF2	Managing finances	8.6	70.6	20.6	8.2	0.6	1: Information; floor effect
LF3	Organizing administration	11.3	63.0	24.2	11.5	1.2	
LF4	Finding a suitable house	32.8	54.4	20.8	23.2	1.6	1: Missing responses; low information
LF5	Organizing the house	20.4	77.0	19.6	2.7	0.7	
LF6	Getting the right amount of light	14.5	69.2	18.9	11.3	0.6	2: Monotonicity
LF7	Living independent	37.6	62.9	25.0	10.3	1.7	
LF8	Organizing the day	2.7	74.0	18.2	7.7	0.0	1: Floor effect; comments YA
HH1	Shopping	3.8	57.5	27.9	11.2	3.4	3: Similar curves HH2 & HH3
HH2	Doing groceries	4.8	55.9	29.9	12.4	1.7	
HH3	Cooking	7.5	58.7	29.1	10.5	1.7	4: Local dependence
HH4	Finding kitchen equipment	1.6	76.5	20.2	2.7	0.5	1: Floor effect; local dependence
HH5	Finding suitable recipes	10.2	76.0	16.2	7.2	0.6	
HH6	Doing household activities	11.3	58.8	29.1	10.9	1.2	
HH7	Operating household devices	1.6	74.3	20.2	4.4	1.1	
SC1	Picking clothes	0.5	80.5	15.7	1.6	2.2	
SC2	Taking care of face	2.2	65.4	27.5	6.0	1.1	1: Information
SC3	Taking care of nails	2.2	56.0	21.4	17.6	4.9	2: Information; H coefficient
SC4	Taking care of hair	1.1	84.8	13.6	1.1	0.5	1: Information; floor effect
SC5	Distinguishing care products	0.0	89.8	9.1	0.5	0.5	
SC6	Taking the right medication	18.8	93.4	5.3	0.0	1.3	1: Information; floor effect
SC7	Following the latest trends	15.6	80.3	14.0	3.8	1.9	1: Floor effect; comments YA
LT1	Gaming	25.3	74.8	12.2	7.2	5.8	1: Missing responses; information; floor effect; local dependence
LT2	Sporting	9.1	55.6	33.7	7.7	3.0	
LT3	Making music	41.4					0: Missing responses
LT4	Going to a cinema, theater or concert	8.1	54.4	28.1	12.9	4.7	
LT5	Following a film in a cinema	7.5	53.5	34.3	9.3	2.9	
LT6	Following a concert or show in a theater	8.6	43.5	36.5	15.9	4.1	
LT7	Following TV series	5.4	67.0	26.1	6.8	0.0	4: Local dependence
LT8	Reading books	4.3	55.6	29.8	11.8	2.8	2: Information; H coefficient
LT9	Finding a suitable sport	9.7	54.2	21.4	22.6	1.8	3: Local dependence; inter-item correlation LT10 & LT11; comments YA
LT10	Participating at a sport club	26.9	48.5	26.5	20.6	4.4	
LT11	Exploring possibilities for sport	11.8	64.0	19.5	14.6	1.8	
HG1	Booking holidays	25.8	72.5	14.5	8.0	5.1	4: Local dependence
HG2	Travelling internationally	15.6	58.0	24.2	14.0	3.8	
HG3	Finding an agreed bar	10.2	48.5	29.9	16.8	4.8	
HG4	Moving in a bar	10.2	42.5	33.5	19.8	4.2	2: Monotonicity; inter-item correlation HG7; comments YA
HG5	Planning a daytrip	2.2	76.4	20.3	2.2	1.1	
HG6	Going on a daytrip	1.6	72.1	20.8	6.0	1.1	
HG7	Participating in nightlife	13.4	46.0	28.6	19.9	5.6	
HG8	Sitting on a terrace	4.8	79.1	17.5	2.8	0.6	4: Local dependence; inter-item correlation HG9
HG9	Going on a weekend trip	5.4	72.7	18.8	6.3	2.3	
HG10	Going on holiday with friends	17.2	72.7	16.2	6.5	4.5	3: Similar curves HG9; inter-item correlation HG9
HG11	Choosing a dish in a restaurant	0.0	53.8	28.5	14.5	3.2	
SR1	Getting to know others	0.5	49.2	40.0	10.8	0.0	2: Local dependence; inter-item correlation SR7
SR2	Making contact with others	2.7	42.0	37.6	18.8	1.7	1: Information; local dependence
SR3	Initiating and maintaining social contacts	0.5	54.1	35.7	10.3	0.0	4: Local dependence
SR4	Recognizing persons	0.0	25.3	43.0	29.6	2.2	2: Information; local dependence
SR5	Recognizing facial expressions	2.2	29.7	26.9	29.7	13.7	3: Information; local dependence
SR6	Getting to know neighbors	5.4	69.3	20.5	9.7	0.6	3: Similar curves SR9
SR7	Making new friends	0.5	49.2	33.5	17.3	0.0	
SR8	Participating in student life	29.0	53.8	25.8	15.9	4.5	4: Local dependence
SR9	Participating in activities	0.5	65.6	28.0	5.9	0.5	
SR10	Meeting at friends	2.2	73.1	22.0	4.4	0.5	3: Local dependence; similar curves SR9 & SR11; inter-item correlation SR9
SR11	Inviting friends	2.7	78.5	16.0	5.5	0.0	3: Local dependence; similar curves SR9 & SR10
RR1	Dating	23.1	51.0	25.2	20.3	3.5	
RR2	Initiating and maintaining a relationship	26.3	60.6	24.8	10.9	3.6	1: Missing responses; information; local dependence; comments YA
RR3	Handling intimacy and sexuality	23.1	71.3	16.8	9.8	2.1	1: Missing responses; information; floor effect; local dependence; comments YA
PC1	Getting into contact with peers	29.0	61.4	26.5	12.1	0.0	1: Missing responses; information; local dependence
PC2	Exchanging experiences with peers	28.0	68.7	22.4	8.2	0.7	1: Missing responses; information; local dependence
PC3	Exchanging experiences regarding visual aids	34.9	71.1	20.7	7.4	0.8	1: Missing responses; information; floor effect; local dependence
PC4	Finding organized activities	40.9					0: Missing responses
PC5	Finding organized holidays	56.5					0: Missing responses
PC6	Exchanging information about work	40.3					0: Missing responses
CO1	Expressing in words	0.5	73.5	21.6	4.9	0.0	1: Information; floor effect; local dependence; monotonicity; comments YA
CO2	Asking questions	0.5	81.1	15.1	3.8	0.0	1: Information; floor effect; local dependence; comments YA
CO3	Expressing feelings	1.1	48.4	34.2	16.8	0.5	2: Information; comments YA
CO4	Participating in a conversation	0.0	65.1	29.6	5.4	0.0	1: Information; local dependence
CO5	Asking acquaintances for help	0.0	64.0	29.0	6.5	0.5	
CO6	Asking strangers for help	0.5	38.9	38.4	20.0	2.7	2: Information; local dependence
CO7	Estimating the emotions of others	0.0	52.2	33.3	14.5	0.0	
CO8	Estimating physical distance to others	1.1	59.8	31.0	8.2	1.1	
CO9	Sharing opinions	0.0	71.5	21.5	7.0	0.0	1: Information; floor effect; local dependence; monotonicity; comments YA
CO10	Expressing what one can and cannot see	1.1	49.5	32.6	16.8	1.1	2: Information; local dependence
CO11	Explaining consequences of visual impairment	0.5	60.5	23.2	15.1	1.1	1: Information; local dependence
IR1	Arranging allowances	18.8	46.4	29.8	21.2	2.6	
IR2	Participating in workshops about study/work	36.6	59.3	23.7	16.1	0.8	
IR3	Participating in online workshops about study/work	51.1					0: Missing responses
IR4	Attending information sessions about studying	53.8					0: Missing responses
IR5	Getting informed about possibilities for studies	31.7	45.7	32.3	19.7	2.4	1: Missing responses; information; local dependence
IR6	Participating in meetings with potential employers	39.8					0: Missing responses
IR7	Finding work with appreciation	25.8	30.4	31.2	33.3	5.1	
IR8	Participating in a job fair	59.1					0: Missing responses
IR9	Finding support for job seeking	30.1	55.4	26.2	17.7	0.8	1: Missing responses; information; local dependence
IR10	Finding information about legislation regarding work	23.7	54.2	26.8	16.9	2.1	4: Local dependence
IR11	Participating in information meetings about legislation	46.8					0: Missing responses
IR12	Participating in a career investigation	36.6	57.6	25.4	13.6	3.4	1: Missing responses; information; local dependence
ST1	Finding a suitable study	11.8	51.2	28.0	20.7	0.0	
ST2	Following classes	12.9	51.2	38.9	9.9	0.0	
ST3	Following study materials	12.9	47.5	39.5	13.0	0.0	3: Local dependence; similar curves ST2
ST4	Participating in courses about computer programs	32.8	64.0	17.6	18.4	0.0	3: Similar curves ST5
ST5	Regulating issues around a study	10.8	68.1	21.7	9.6	0.6	
ST6	Following a study	13.4	62.7	26.1	10.6	0.6	
ST7	Doing homework	11.8	64.0	26.8	9.1	0.0	4: Local dependence
ST8	Finishing a study	19.9	53.7	30.2	16.1	0.0	1: Information; local dependence
AP1	Applying	16.7	56.8	31.6	9.7	1.9	
AP2	Presenting during a job interview	15.1	52.5	34.8	12.0	0.6	2: Local dependence
AP3	Knowing who to approach for employers’ questions	22.0	60.0	23.4	14.5	2.1	1: Missing responses; information; local dependence; comments YA
AP4	Telling employer about visual impairment	14.5	43.4	26.4	29.6	0.6	2: Information; local dependence
WO1	Recognizing colleagues	15.6	52.9	34.4	12.7	0.0	
WO2	Having a paid job	28.0	53.0	19.4	19.4	8.2	1: Missing responses; information; local dependence
WO3	Having voluntary work	51.1					0: Missing responses
WO4	Handling work pressure	15.6	45.2	38.9	14.6	1.3	
WO5	Presenting in business environment	17.2	59.1	31.8	9.1	0.0	
WO6	Performing work adequately	17.7	68.6	26.8	3.9	0.7	
AS1	Understanding visual impairment	0.0	70.4	22.6	7.0	0.0	1: Information; floor effect; local dependence
AS2	Feeling confident	0.0	31.2	46.2	21.5	1.1	
AS3	Presenting in private environment	0.5	67.0	25.4	7.6	0.0	3: Local dependence; similar curves AS2 & AS4
AS4	Maintaining energy levels	0.0	36.0	38.7	23.7	1.6	
AS5	Preventing feelings of loneliness	3.2	48.3	36.1	13.3	2.2	
AS6	Feeling equal to others	1.1	44.0	35.3	15.8	4.9	1: Information; local dependence; comments YA
AS7	Dealing with misunderstanding	12.4	40.5	33.1	24.5	1.8	

RV: reading and visual aids; MO: mobility; CS: computer skills; LF: living independent and finances; HH: household; SC: self-care; LT: leisure time; HG: holiday and going out; SR: social relationships; RR: intimate/romantic relationships; PC: peer contact; CO: communication; IR: information/regulations; ST: study; AP: applying; WO: work; AS: acceptance/self-consciousness

^a^ item content is not an official translation

Explanation of phases

0: removed because >80 missing responses before primary analysis

1: removed after the 1^st^ IRT analysis because of missing responses, floor effects, low information, no monotonicity, local dependence and/or comments of YA

2: removed after the 2^nd^ IRT analysis because of low information, no monotonicity, low H-coefficient, local dependence, inter-item correlation and/or comments of YA

3: removed after the 3^rd^ IRT analysis because of low information, local dependence, inter-item correlation, similarity in curves (ICCs, CRCs, IICs) and/or comments of YA

4: removed after the 4^th^ IRT analysis because of local dependence and/or inter-item correlations

#### IRT assumptions

The acceleration factor suggested the PAI-YA consisted of one latent dimension, i.e. participation. PCA components for the one-factor solution were all positive and mostly acceptable. Inspection of item and factor content gave no reason for multidimensional solutions; a two-factor solution did not substantially add to the explained variance (29% vs. 34%). Based on these results, the 128 items comprised a unidimensional scale which is sufficient for IRT analysis. However, excess item covariation (>0.25) among 102 item pairs indicated a violation of the local independence assumption. Initially, this was not considered to be a problem, since IRT models are often robust to violations of local dependence, especially when the scale consists of >10 items [47]. Although items which formed questionable pairs were considered candidates for deletion, actual deletion was done with reluctance; the least performing item was selected. Monotonicity analysis showed that two items were not monotonically increasing and scalability analysis showed that 21 items had an H coefficient below 0.3 (Table 2). These items were considered candidates for elimination.

#### IRT analysis

Five iterations of item selection and IRT analysis were conducted to identify items to be removed due to low information, weak curves or subsequent violations of the IRT assumption. A total of 68 items were deleted, 34 in the first iteration, 12 in the second, 11 in the third and 11 in the fourth iteration, resulting in a 60-item PAI-YA. Table 2 shows the distribution of responses and reasons for item removal. Item information and less violations in assumptions occurred at each iteration, although in the 60-item PAI-YA two items violated the monotonicity assumption (CS8 and LT10) and one item had a H coefficient of 0.29 (SC5). Excess covariation remained among six item pairs (RV1-RV2, RV5-CS4, MO5-MO6, CS5-CS8, SR7-RR1, ST2-ST6), violating the assumption of local independence. Despite (small) violations in assumptions, further item reduction was considered to be unfavorable for maintaining content validity. The Likelihood Ratio Test indicated that the full GRM improved fit upon the polytomous Rasch model (LRT value = 162.36, df = 59, p = 0.001). Item information for the 60 items ranged from 1.40 to 4.66, and none of the items contributed <0.75% to the total information (155.42).

### Psychometric properties of the PAI-YA

**Fig 1:**
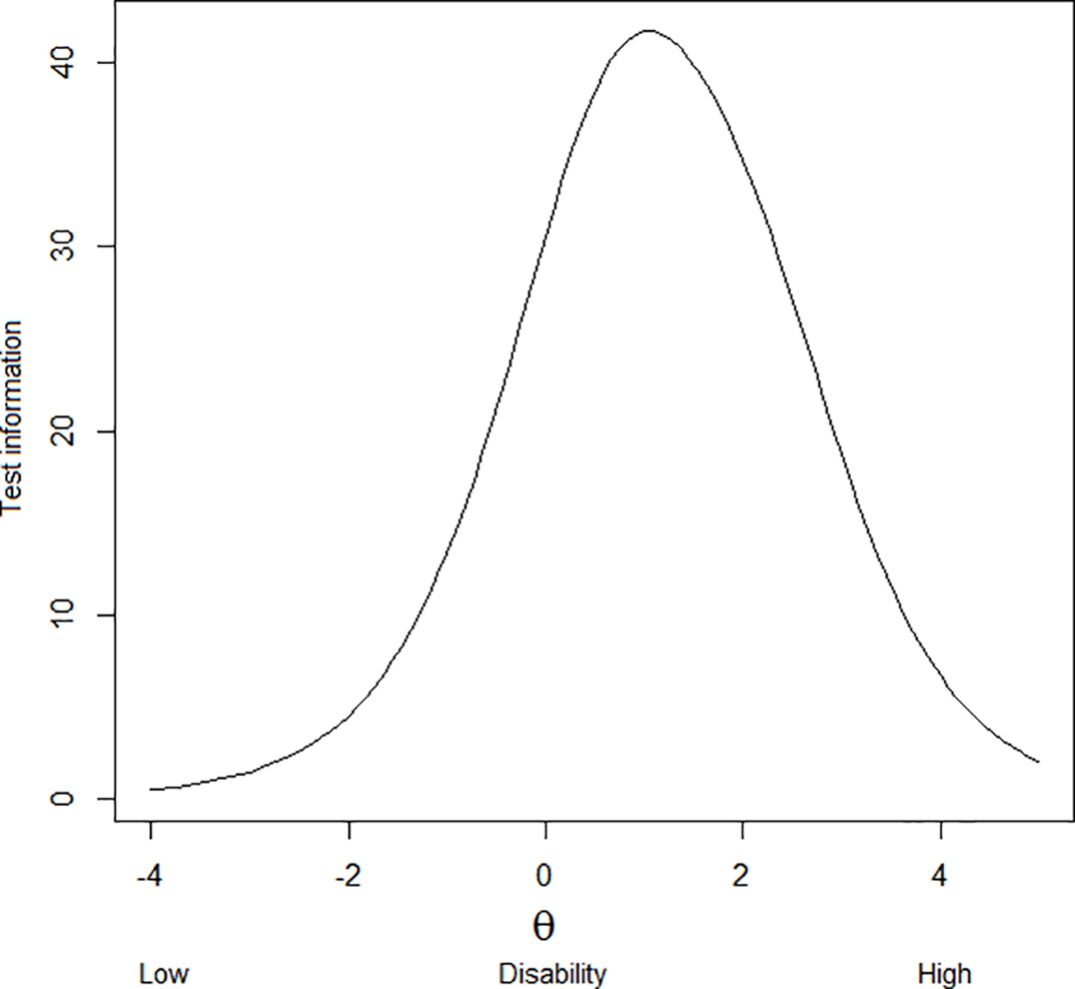
Test information curve of the PAI-YA.

**Fig 2:**
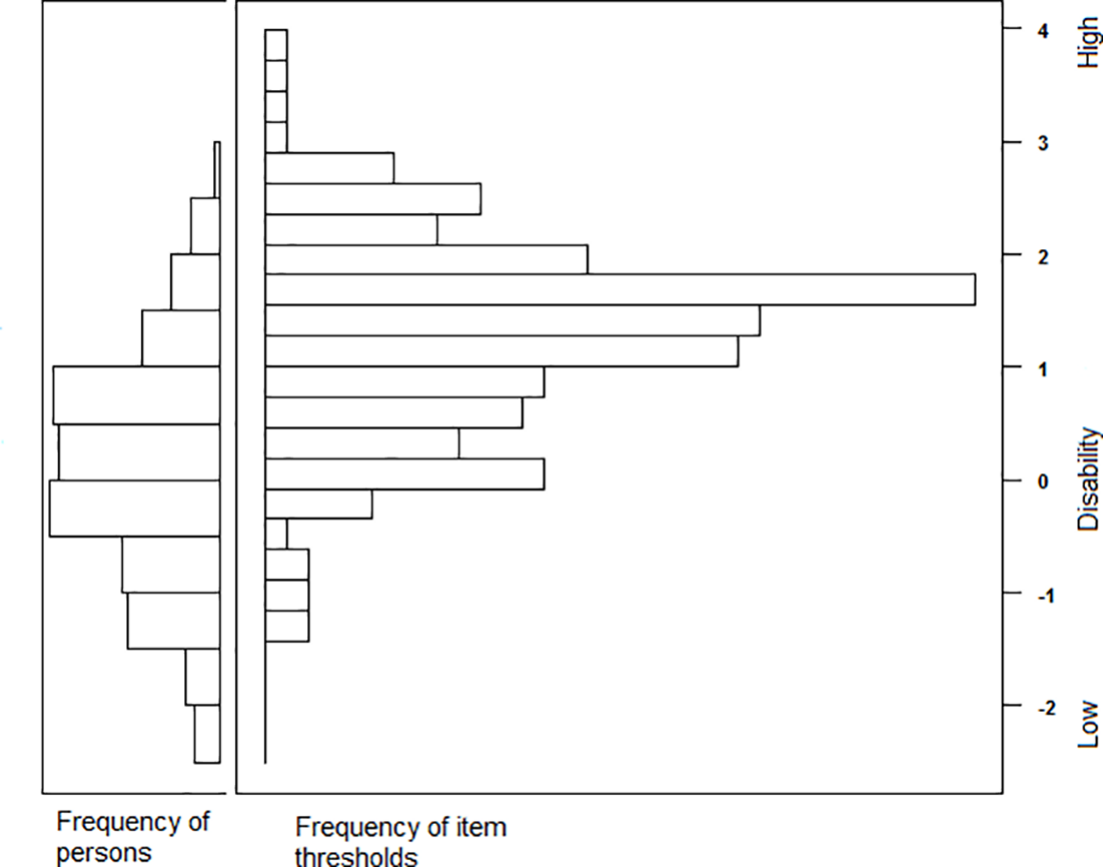
Item-person map of the PAI-YA.

The fit indices reflected an adequate overall model fit of the 60 items: RMSEA = 0.057, SRMR = 0.072, CFI = 0.967 and TLI = 0.966. However, the M2 statistic was significant (2662.32 ± 88.06, p<0.05). Fig 1 presents the test information curve of the PAI-YA, providing information about its precision across the disability continuum. The PAI-YA seemed less precise at the extremes, but covered most of the disability continuum; however, the lower extreme was not entirely covered. Table 3 presents the parameter estimates for the remaining 60 items of the PAI-YA. Note that the items have been restructured into new or existing domains, as some domains only included a limited number of items after item removal, or even none. Restructuring of items was done based on the results of the concept-mapping study used to design the PAI-YA [5]. The slope estimates ranged from 0.84 to 2.61, indicating considerable variation in item discrimination. The location parameters for the 60 items reflect a sizeable range of underlying disability (-1.40 to 3.73), but the majority of item response categories were only endorsed by respondents who had higher than average levels of disability (i.e. θ >0), indicating that the PAI-YA as a whole is most useful in discriminating among individuals at the high end of the disability continuum. This is also shown in Fig 2, which shows the item-person map of the PAI-YA; items are distributed across the largest part of the disability continuum. The distribution of patients’ scores matches the distribution of items adequately, although there are no items to discriminate between patients at the low end of the disability continuum. ANOVA with post-hoc Tukey tests showed that persons with mild vision loss had significantly lower thetas than persons with low vision or who were blind (p = 0.001 and p<0.001, respectively), indicating that they were less disabled and the PAI-YA was able to discriminate between them. Furthermore using ANOVA and independent samples t-tests, significantly lower thetas were found for financial situation (lower theta for usually money left vs. not enough money, p = 0.016), mode of administration (lower theta for online vs. paper-and-pencil, and lower theta for telephone interview vs. paper-and-pencil, p = 0.009 and p = 0.022, respectively), males (p = 0.014), participants who did not have a cognitive impairment (p = 0.035), who were currently studying (p = 0.001), and who currently had a paid job (p = 0.005). A significant positive correlation was found for theta and age (p = 0.001). To correct for other variables, multiple linear regression showed significant associations between thetas and degree of visual impairment (p = 0.001), financial situation (p = 0.028) and sex (p = 0.029). Internal consistency for the LVQOL and subscales of the SF-36 and IPA was good, with Cronbach’s alpha of >0.7 (Table 4). As expected, correlations between the PAI-YA and (scales of) the SF-36, EQ-5D and LVQOL were negative and those between the PAI-YA and scales of the IPA were positive. The PAI-YA was significantly correlated with (all scales of) the SF-36, EQ-5D, IPA and LVQOL (p<0.01). The strength of the correlations were within the expected range, except for the SR and WO scales of the IPA, which were expected to correlate moderately with the PAI-YA but showed strong correlations instead (Table 4). Test-retest reliability of the PAI-YA items was satisfactory for all items (Table 3). In total, 11 items had moderate kappa values (>0.40), 46 had good values (>0.60) and 3 items had very good values (>0.80). On inspection, 31 items had moderate agreement (60–74%), 28 had good agreement (75–89%) and 1 item had excellent agreement (≥90%).

**Table 3:** GRM item parameter estimates and item information for the 60-item PAI-YA (n = 186).

Item	Item content^a^	Discrimi-nation α	Extremity β1	Extremity β2	Item information	Agreement %	Weighted kappa
**Reading, visual aids and computer skills**							
RV1	Reading text	1.00	-0.32	2.03	1.66	70.5	0.66
RV2	Reading handwriting	1.20	-1.33	0.69	2.02	79.4	0.81
RV5	Using visual aids	1.20	0.83	2.90	2.02	71.6	0.50
CS4	Finding suitable computer software	1.79	0.58	1.63	2.79	68.5	0.52
CS5	Using the computer	1.25	1.69	3.73	2.12	83.2	0.62
CS8	Using social media	1.57	1.79	2.76	2.31	87.3	0.69
**Mobility**							
MO1	Walking	1.64	0.97	2.32	2.68	82.7	0.75
MO2	Cycling	1.81	0.01	1.02	2.81	83.7	0.87
MO5	Participating in traffic at daytime	1.55	0.48	2.34	2.73	73.0	0.64
MO6	Participating in traffic at night	0.84	-1.40	1.38	1.40	70.3	0.68
MO7	Estimating the speed of other traffic	1.16	-1.03	1.27	1.99	72.6	0.65
MO10	Traveling with public transport	1.72	0.19	1.73	2.95	79.6	0.76
MO12	Finding the way to/at an unknown location	2.10	-0.68	0.58	3.60	72.5	0.78
MO15	Finding the way to school or work	2.55	1.15	2.50	4.66	88.2	0.71
MO16	Finding the way at school and work	1.66	0.97	2.55	2.85	82.0	0.62
**Living independently and finances**							
IR1	Arranging allowances	1.28	0.03	1.38	1.95	67.8	0.69
LF1	Paying	1.64	1.10	3.00	2.94	79.3	0.55
LF3	Organizing administration	1.76	0.56	1.73	2.81	77.8	0.70
LF5	Organizing the house	1.93	1.11	2.63	3.40	83.3	0.63
LF7	Living independent	1.83	0.62	1.83	2.99	81.6	0.79
**Household and self-care**							
HH2	Doing groceries	1.60	0.36	1.75	2.62	83.3	0.83
HH5	Finding suitable recipes	1.41	1.22	2.41	2.14	85.3	0.70
HH6	Doing household activities	1.74	0.38	1.77	2.92	77.7	0.75
HH7	Operating household devices	1.97	0.97	2.25	3.34	82.4	0.57
SC1	Picking clothes	1.52	1.38	2.83	2.49	84.6	0.60
SC5	Distinguishing care products	1.61	1.97	3.63	2.76	92.0	0.55
**Sport and leisure time**							
LT2	Sporting	1.78	0.27	1.86	3.10	67.4	0.58
LT4	Going to a cinema, theater or concert	1.78	0.21	1.38	2.86	82.8	0.78
LT5	Following a film in a cinema	1.73	0.17	1.75	2.99	65.1	0.62
LT6	Following a concert or show in a theater	1.24	-0.27	1.49	2.01	69.0	0.64
LT10	Participating at a sport club	1.64	0.04	1.09	2.49	66.0	0.63
LT11	Exploring possibilities for sport	1.86	0.56	1.43	2.79	78.1	0.76
**Holiday and going out**							
HG2	Travelling internationally	1.93	0.32	1.30	3.02	75.7	0.80
HG3	Finding an agreed bar	2.38	0.01	1.04	4.00	77.5	0.79
HG5	Planning a daytrip	1.96	1.08	2.66	3.51	86.2	0.63
HG6	Going on a daytrip	2.41	0.83	1.96	4.17	84.3	0.76
HG7	Participating in nightlife	1.54	-0.16	1.04	2.38	75.2	0.76
HG9	Going on a weekend trip	2.61	0.80	1.75	4.41	80.9	0.62
HG11	Choosing a dish in a restaurant	1.34	0.24	1.60	2.07	68.7	0.68
**Social relationships**							
SR7	Making new friends	1.34	0.05	1.64	2.16	71.8	0.70
SR9	Participating in activities	1.60	0.68	2.38	2.77	70.4	0.47
RR1	Dating	1.05	0.02	1.35	1.51	73.0	0.72
CO5	Asking acquaintances for help	0.89	0.83	3.35	1.46	72.0	0.54
CO7	Estimating the emotions of others	0.99	0.15	2.17	1.57	75.4	0.72
CO8	Estimating physical distance to others	1.31	0.47	2.33	2.19	73.0	0.62
**Study**							
ST1	Finding a suitable study	1.24	0.09	1.45	1.88	70.2	0.71
ST2	Following classes	1.36	0.06	2.13	2.37	74.9	0.69
ST5	Regulating issues around a study	1.82	0.69	1.83	2.91	73.5	0.67
ST6	Following a study	2.02	0.47	1.68	3.41	74.8	0.65
IR2	Participating in workshops about study/work	2.52	0.42	1.35	4.18	64.8	0.56
**Work**							
AP1	Applying for work	1.29	0.27	2.01	2.12	68.3	0.51
IR7	Finding work with appreciation	1.67	-0.69	0.53	2.67	63.1	0.60
WO1	Recognizing colleagues	1.05	0.11	2.21	1.71	81.7	0.80
WO4	Handling work pressure	1.46	-0.21	1.58	2.50	73.6	0.68
WO5	Presenting in business environment	1.13	0.39	2.45	1.87	73.5	0.56
WO6	Performing work adequately	1.48	0.74	2.64	2.57	79.0	0.66
**Acceptance and self-consciousness**							
AS2	Feeling confident	0.93	-0.95	1.67	1.56	73.4	0.72
AS4	Maintaining energy levels	1.11	-0.60	1.31	1.78	68.0	0.66
AS5	Preventing feelings of loneliness	1.18	0.03	1.92	1.93	72.6	0.70
AS7	Dealing with misunderstanding	1.02	-0.34	1.35	1.56	64.0	0.61

^a^ item content is not an official translation

**Table 4:** Correlation coefficients of the PAI-YA with (scales of) other instruments.

	Expected correlation PAI-YA	N	Correlation PAI-YA	Cronbach’s alpha
SF-36 scales				
PF	Moderate/strong -	171	-0.39*	0.90
SF	Moderate/strong -	171	-0.47*	0.83
RP	Moderate/strong -	171	-0.51*	0.79
RE	Moderate -	171	-0.33*	0.83
MH	Moderate -	171	-0.43*	0.86
VT	Moderate -	171	-0.34*	0.79
BP	Moderate -	171	-0.35*	0.79
GH	Moderate/strong -	171	-0.53*	0.85
EQ-5D	Strong -	170	-0.53*	n.a.
IPA scales				
AI	Moderate/strong +	169	0.34*	0.91
FR	Moderate/strong +	169	0.57*	0.92
AO	Moderate/strong +	169	0.67*	0.85
SR	Moderate +	169	0.52*	0.83
WO	Moderate +	110	0.72*	0.89
LVQOL	Strong -	163	-0.69*	0.90

*Significant correlation (p<0.01)

+ positive correlation

- negative correlation

n.a. not applicable

### Evaluation and optimized version of the PAI-YA

Over 90% of the young adults were neutral to positive on several aspects of the PAI-YA (Table 5). Mean self-reported administration time of the PAI YA (including questions on demographic and clinical characteristics) was 45.52 ± 27.90 (range 8–180, median 40) min. After removal of the 81 items, it was assessed whether all rehabilitation questions young adults asked could still be identified with the 60-item PAI-YA. This was possible with the exception of rehabilitation questions related to having difficulty explaining the consequences of visual impairment to others (CO11), getting into contact with peers (PC1), driving a car (MO4) and taking care of nails (SC3). Therefore, it was decided to include these items in the PAI-YA, but not to score them because they are not part of the unidimensional scale. Moreover, young adults suggested to add another response option between ‘slightly difficult’ and ‘very difficult’; the response option ‘difficult’ was added. The PAI-YA thus consists of 64 questions which can be scored on a 4-point Likert scale with response options: not difficult (1), slightly difficult (2), difficult (3) and very difficult/impossible (4).

**Table 5:** Evaluation of the PAI-YA by young adults (N = 186).

Meaningfulness PAI-YA for insight in possibilities of rehabilitation	
Meaningless, N (%)	1 (0.5)
Not meaningful, N (%)	4 (2.2)
Neutral, N (%)	42 (22.6)
Meaningful, N (%)	105 (56.5)
Very meaningful, N (%)	34 (18.3)
Representation of commonly experienced challenges in PAI-YA	
Bad, N (%)	5 (2.7)
Moderate, N (%)	9 (4.8)
Reasonable, N (%)	49 (26.3)
Good, N (%)	88 (47.3)
Very good, N (%)	35 (18.8)
Difficulty choosing the appropriate response option in the PAI-YA	
Always/almost always, N (%)	3 (1.6)
Often, N (%)	10 (5.4)
Regularly, N (%)	26 (14.0)
Sometimes, N (%)	89 (47.8)
Never/almost never, N (%)	58 (31.2)
Satisfaction with administration time of the PAI-YA	
Very unsatisfied, N (%)	1 (0.5)
Unsatisfied, N (%)	10 (5.4)
Neutral, N (%)	41 (22.0)
Satisfied, N (%)	102 (54.8)
Very satisfied, N (%)	32 (17.2)

## Discussion

In this study, psychometric properties were investigated to improve the PAI-YA: a questionnaire to identify and monitor the needs of young adults with a visual impairment. Following a developmental and piloting phase, this study led to item reduction and assessed the psychometric properties of the remaining items of the PAI-YA. The PAI-YA has good psychometric properties and covers a broad range of aspects of measuring the participation of young adults aged 18–25 years with a visual impairment. Because a national sample was used to assess its properties, the PAI-YA should be applicable across the Dutch population of young adults aged 18–25 years with visual impairment from any cause. Although the PAI-YA can serve as a template for use in other countries and languages, cross-cultural validation is recommended for use outside the Netherlands.

The item reduction process followed a rigorous strategy, combining aspects from classical test theory (CTT) and IRT, while also considering content validity because it should cover broad aspects of participation. IRT has added value in the process taken to reach conclusions, as it contains detailed item-level information, and its insights are most useful when complemented by results from CTT [13] and the target group’s perspective, as was done in this study. This comprehensive strategy led to the removal of 81 items, and re-inclusion of four of these items, resulting in a PAI-YA of 64 items (but only 60 are used in the scoring of the unidimensional scale).

The IRT assumptions seemed to hold for most of the 60 items. PCA showed that the PAI-YA consisted of one factor, as confirmed by the acceleration factor. Since IRT analysis requires a unidimensional scale, these results were important for continuation of the analyses. Although Kaiser’s eigenvalue >1 and Cattell’s scree plot methods are mostly used to determine the number of factors [65], simulation studies suggest that the acceleration factor outperforms these methods in determining the number of factors [66, 67]. There were six item pairs with local dependence, and the monotonicity assumption was violated for two items. However, a compromise had to be made between the aim of this study and the overall aim of the PAI-YA, i.e. to develop an instrument with strong psychometric properties versus developing a feasible instrument which can be used to investigate a broad range of rehabilitation needs to develop a rehabilitation plan. Therefore, content representation was carefully considered and further item reduction was deemed unfavorable.

The full GRM fitted the PAI-YA data well and all fit indices were satisfactory, although M2 was significant. The good dispersion of the discrimination parameters reaffirmed that the full GRM would better fit the PAI-YA data than the constrained GRM model, which assumes that all items have equal discrimination. The extremity parameters reflected a broad range of underlying disability for the 60 items. Standard errors of the discrimination and first extremity parameters were generally small (mostly 0.1–0.2 for the location parameters and 0.1–0.3 for the discrimination parameters), suggesting that these parameter estimates were relatively precise. However, standard errors for the second extremity parameter were much higher, which is caused by the infrequent endorsement of the response option ‘very difficult/impossible’ and the low number of participants. Although there are no definite answers regarding sample size requirements, general guidelines are available. For example, Tsutakawa and Johnson recommend a sample size of approximately 500 for accurate parameter estimates [68], whereas others suggest that 200 participants or less can be adequate [69, 70]. Nevertheless, large standard errors are considered less problematic when evaluating questionnaire properties, as in this study, compared to estimating accurate person measures (thetas).

The PAI-YA seemed better targeted to young adults having thetas at the higher end of the disability continuum. As shown by the item-person map, persons with low disability were less well discriminated by items of the PAI-YA, and there might be a need for more difficult items. This is also found in studies evaluating the psychometric properties of questionnaires intended for visually impaired children [71, 72]. However, the target population of the PAI-YA does not entirely match the population of respondents included in the present study, as all these participants were currently or previously enrolled for care at an MRC and are likely to have received rehabilitation services in the past. Although IRT models should be quite robust to differences in population characteristics [73], this may have slightly biased the results, as this group might not experience difficulties in participation and activities to the same extent as young adults with a visual impairment who seek rehabilitation services.

The PAI-YA is intended to investigate the rehabilitation needs of young adults aged 18–25 years with normal cognitive functioning who seek low vision rehabilitation services. Despite the inclusion criteria of our study, seven participants did not meet the age criterion of the PAI-YA, and eight participants reported to be cognitively impaired. Removing these participants from the dataset did not alter the item parameters pattern (data not shown). For six items the extremity β2 parameter changed more than 0.5 on the disability continuum (theta). However, all items except CS5 ‘using the computer’, which shifted to the left on the disability continuum (i.e. becoming more difficult), were removed from the PAI-YA during the first iterations of the psychometric analyses. Therefore inclusion of these participants did not have a large influence on the outcomes of this study.

The results of this study support the construct validity of the PAI-YA, having a unidimensional structure. Known-group validity showed significant differences in thetas for visual impairment, mode of administration, age, financial situation, sex, educational situation and employment situation. In most cases, the differences between these groups were expected. Although women more often score worse than men on quality of life instruments, and worse scores associated with increasing age were seen in a study on quality of life in visually impaired children [38, 43, 74], the differences between males and females and the differences between age groups were not expected. Because the difference found in mode of administration was based on only two participants who filled in the questionnaire using a paper-and-pencil version, this finding is likely to be distorted. Although a difference was expected between persons with and without comorbidity, this was not found (p = 0.089). Significant associations between thetas and severity of visual impairment, financial situation and sex remained in a multiple linear regression model, after correction for all other variables.

Concurrent validity was established by relating PAI-YA scores to scores of the SF-36, EQ-5D, LVQOL and IPA, with the expected correlations found. To our knowledge, the SF-36 and EQ-5D have not been validated specifically in a visually impaired population, although Malkin et al. investigated the responsiveness of the EQ-5D in a sample of participants with a visual impairment prior to and after rehabilitation [75]. They concluded that the EQ-5D is unresponsive as outcome measure for low vision rehabilitation and has poor sensitivity for discriminating people with a visual impairment with different levels of ability. However, both the EQ-5D and SF-36 have extensively been used across a range of populations and diseases, including visually impaired populations and ophthalmic conditions (e.g. [2, 76–78]), which makes the instruments suitable for comparisons.

Despite the long time-interval between completion of test and retest for some of the participants (i.e. less than 5% of the participants completed the retest at least 3 months after completion of the test), test-retest reliability was satisfactory; moderate to very good kappa values and moderate to excellent agreement was found in all items. Excluding participants who completed the retest more than a month after the first measurement only had minimal influence on kappa values and agreement. In 32 items the kappa values improved (two from moderate to good, three from good to very good), whereas in 21 items kappa values deteriorated (one from very good to good, five from good to moderate). The largest difference was found for item SR9 ‘participating in activities’, with an improvement from 0.47 to 0.55. Agreement slightly improved in 23 items and deteriorated in 37 items, however, differences were no larger than 5%. The large number of instruments and the length of the 141-item PAI-YA used in this study might have affected the quality of the data and might have introduced acquiescence bias, i.e. the tendency to opt for the same answer regardless of the content of an item [79]. However, no indications for acquiescence bias were found, as variability in responses was observed throughout the questionnaire and the number of missing responses did not increase towards the end of the questionnaire. Furthermore, 85% of the participants completed all instruments, and participants were informed before the start of the study on the expected duration to fill in the questionnaires.

The items of the PAI-YA are formatted on a 4-point Likert scale, which makes the questionnaire easy to administer. An online survey questionnaire should be the preferred mode of administration, because it is least susceptible to errors or leaving items blank, and is least time consuming to employees of MRCs. However, if filling in an online survey questionnaire is not feasible for the young adult because of the visual impairment or other reasons, a telephone interview is the second preferred option. Although young adults were mostly neutral to positive about various aspects of the 141-item PAI-YA, including time to complete, the administration time is expected to significantly reduce with item deletion. Since the PAI-YA is planned to be used by Dutch low vision MRCs, this will make use of the PAI-YA more feasible. When the PAI-YA is used in rehabilitation practice, it will be possible to re-assess the psychometric properties of the PAI-YA because of increased response rates. This will enable further item reduction, resulting in a shorter, more precise and user-friendly instrument. Besides rehabilitation practice, the PAI-YA might also be suitable for use in research investigating participation levels of visually impaired young adults.

Future research should examine whether the model still fits the data after the addition of the response option ‘difficult’. Moreover, differential item functioning (DIF) could not be assessed because of the small sample size. In addition, responsiveness of the PAI-YA should be investigated, which may lead to some more adaptations. In future, a computerized adaptive test (CAT) to tailor the PAI-YA to the abilities of visually impaired young adults might be developed and evaluated. This application might be particularly useful to monitor the personal needs and goals of visually impaired young adults, as it ensures that young adults do not have to fill in the full PAI-YA multiple times. Furthermore, the latent disability scores of young adults, which are preferred over sum scores because of their ability to handle missing data, might in future be made immediately available to rehabilitation services providers. This could help improve patient-centered care by increasing communication between young adults and MRC professionals, enhancing MRC professionals’ understanding of the needs of young adults, and facilitating the integration of young adults’ perspectives in their rehabilitation process. Moreover, it is expected that the PAI-YA will help to structure the intake procedure at low vision MRCs and can be used to evaluate the effectiveness of rehabilitation.

In conclusion, the results of this study show that the GRM model fits the PAI-YA data well. This study provides detailed information on item parameters and has resulted in a shorter version of the PAI-YA consisting of 64 items. This study provides evidence of construct validity, known-group validity, concurrent validity and test-retest reliability of the PAI-YA. Furthermore, it is an important step in the assessment of psychometric properties of the PAI-YA, and in the process to develop a feasible instrument to investigate and monitor the rehabilitation needs of visually impaired young adults.

## Supporting information

S1 Dataset Dataset used for analyses of the PAI-YA. (XLSX)
